# Impact of NICE guidance on tamoxifen prescribing in England 2011–2017: an interrupted time series analysis

**DOI:** 10.1038/s41416-018-0065-2

**Published:** 2018-04-23

**Authors:** Helen J. Curtis, Alex J. Walker, Ben Goldacre

**Affiliations:** Evidence Based Medicine DataLab, Centre for Evidence Based Medicine, Nuffield Department of Primary Care Health Sciences, University of Oxford, Radcliffe Observatory Quarter, Woodstock Road, Oxford, OX2 6GG United Kingdom

**Keywords:** Breast cancer, Health policy, Cancer prevention

## Abstract

**Background:**

Tamoxifen was recommended by NICE in 2013 for chemoprevention of breast cancer, but a recent survey suggested only a quarter of GPs are aware of this. We set out to measure the uptake of tamoxifen, and the alternative raloxifene, in national prescribing data sets.

**Methods:**

Tamoxifen and raloxifene data were extracted from England’s monthly prescribing data sets, October 2010–October 2017. We used interrupted time series analysis to reveal national and local responses to guidelines. We investigated variation between practices by calculating percentiles for prescribing rates and ratios of change.

**Results:**

We found an increase in monthly tamoxifen prescribing following release of the guidelines, with an increase in gradient (*p* = 0.001) but no step change (*p* = 0.342). Alongside a small change in raloxifene prescribing we estimate 8450 women took up chemoprevention between 2013 and 2016. We did not find evidence that this was limited to a small group of practices.

**Conclusions:**

Our results suggest that the uptake of new guidance on chemoprevention has been slow and has potentially left women exposed to avoidable risk. Improving dissemination of guidance to healthcare professionals and routinely monitoring implementation could help reduce this risk.

## Introduction

Breast cancer is the most common cancer in the UK, with 55,200 diagnoses and over 11,000 deaths in 2014.^1^ Tamoxifen, a Selective Oestrogen Receptor Modulator (SERM), is a well-established adjuvant treatment for breast cancer.^2,3^ It is also used as a preventive intervention for women with increased risk of developing breast cancer, at least 17% during their lifetime, calculated based upon several risk factors including family history.^4^ In this cohort, tamoxifen is estimated to reduce the incidence of breast cancer by over a third.^5,6^

The prescription of daily tamoxifen to women at increased risk, for up to five years, was recommended by the National Institute for Health and Care Excellence (NICE) in June 2013, with raloxifene as an alternative for post-menopausal women. It was recently reported that only 50% of general practitioners (GPs) know of the chemopreventive effect of tamoxifen and 25% are aware of the guidelines.^7^ This is perhaps because discussion of this chemoprevention often takes place in specialist clinics; however, GPs are also expected to initiate appropriate prescribing.^8^ Regardless of where treatment is initiated, most ongoing tamoxifen prescribing in the UK will be taken over by the patient’s GP, and hence be included in primary care prescribing data.

We therefore set out to explore the prescribing pattern of tamoxifen before and after the release of the NICE guidelines using interrupted time series analysis; and to identify whether any change is limited to only a subset of practices, reflecting an incomplete dissemination of evidence as previously described. We also investigated the local effect on trends in prescribing from a single earlier study aiming to increase tamoxifen uptake in one region, to validate that local changes in prescribing over time could be detected in the data used.

## Methods

### Data sources and preparation

The monthly prescribing data sets published by NHS Digital are obtained from pharmacy claims and contain one row for each treatment and dose, in each prescribing organisation in NHS primary care in England, describing the number of prescriptions dispensed and the total cost. From this we extracted the prescribing data on all brands and presentations of tamoxifen and raloxifene (BNF codes beginning 0803041S0 and 0604011X0, respectively) as well as aromatase inhibitors: anastrozole, exemestane and letrozole, for comparison (Table S1, Appendix 1) for the period October 2010–October 2017.

We limited the data to prescriptions issued by general practices (GPs) and excluded all other organisations (such as prisons, out-of-hours services, and other non-standard settings) by selecting only those institutions where the setting is coded as standard primary care in the NHS Digital data set of practice characteristics.^9^ Although tamoxifen prescribing is often initiated in specialist clinics, it will generally be taken over in primary care, thus ensuring that the prescribing data appears in the primary care prescribing data set used here. Practice list sizes with additional data on the age and sex structure of the local population were obtained from NHS Business Service Authority’s Information Portal, and assigned to each individual month’s prescribing by taking the most recent quarter’s figure. Practices were excluded if the number of females registered between the age of 35 and 44 was always below 10; this will exclude most remaining specialist practices or clinics serving non-typical populations, including homeless services, walk-in centres and care homes.

In the prescribing data set, the quantity dispensed per prescription is aggregated for each identical presentation and dose (e.g. one prescription for 30 tamoxifen 20 mg tablets and one for sixty 20 mg tablets becomes two prescriptions for ninety 20 mg tablets in total). We normalised the quantity prescribed of each different presentation to the standard daily dosages of 20 mg (tamoxifen), 60 mg (raloxifene) and as appropriate for each aromatase inhibitor (Table S1, Appendix 1) to calculate the number of average daily quantities (ADQs) prescribed. For each practice, and nationally, we calculated the prescribing rate as the total ADQs prescribed per 1000 registered females between the ages of 35 and 74 (tamoxifen) or 45+ (raloxifene and aromatase inhibitors).

### Interrupted time series analysis (ITSA) for national prescribing

ITSA allows the effect of interventions to be analysed taking into account underlying trends.^10^ We conducted ITSA using monthly prescribing data and adjusted for seasonality by including each calendar month as an independent variable in the model. We set the ‘intervention’ time as June 2013, the publication month of the relevant NICE guidance. We used the Stata ‘itsa’ module, which produces Newey–West standard errors for coefficients estimated by ordinary least-squares regression. ADQs per day were calculated by dividing the monthly rate across a given period by 30 (or by actual number of days in a given month).

### National variation

To assess whether there was variation in practices’ response to the guidance, as suggested by recent research, we calculated ratios of prescribing rates before and after publication, and then graphed deciles of these ratios over time. ADQs per 1000 were averaged over rolling six-month windows, where the data point is given as the final month of the period. Only complete 6-month periods were included, the first being Oct 2010–Mar 2011. Practices were included only during months they prescribed more than zero total items. We also calculated the ratio of the prescribing rate for each window to the baseline period of December 2012–May 2013 (immediately prior to guidance publication), excluding practices prescribing zero tamoxifen items at baseline. We calculated and graphed percentiles for prescribing rates and ratios across all practices at each time point from March 2011 to October 2017.

### Validation of ability to detect local variation

An intervention study ran from May 2011 to November 2012 in Greater Manchester (GM) offering tamoxifen chemoprevention to eligible patients.^11^ This offered an opportunity to investigate local changes in prescribing. Practices located in GM were identified using a list of postcodes obtained from http://www.postcodefinder.org.uk/. For tamoxifen and raloxifene, we carried out two-variable ITSA to compare prescribing at practices in GM with other practices in England. This permitted us to measure change in prescribing between a region which had previously offered tamoxifen and other areas which had not. For context we also plotted prescribing rates for aromatase inhibitors over time for GM practices versus all others.

### Impact of NICE guidance

We estimated the impact of NICE guidance in terms of number of women taking up chemoprevention as indicated by change in number of daily ADQs prescribed. We used this to predict the number of cancers that may be prevented, using numbers-needed-to-treat from clinical trials. We also estimated the maximum total population in England, which could be expected to take chemoprevention if offered, in order to place our results in context of what could theoretically be achievable.

### Data and code

Data were extracted using SQL in Google BigQuery. Decile calculations were carried out in Stata 13 and plotted in Excel. ITSA was carried out and plotted in Stata 13 using the ‘itsa’ module. SQL code is available in Appendix 2 and Stata code in Appendix 3 under MIT license. Complete data sets are provided in Appendices 4–6.

## Results

### Data sources and preparation

We excluded 66 practices due to very low relevant population size (females 35–44 ≤ 10 at all time points). Of the remaining 8090 practices, 875 opened or closed during the study period. We excluded a further 452 practices from the ratios analysis due to having no tamoxifen prescribing during the baseline period; 131 of these were not active as practices during the baseline period. For tamoxifen ITSA, we limited analysis to the period of constant rate post-intervention eligible for analysis (up to December 2016), therefore included 8082 practices: 423 in GM and 7659 others.

### Interrupted time series analysis

**Fig. 1:**
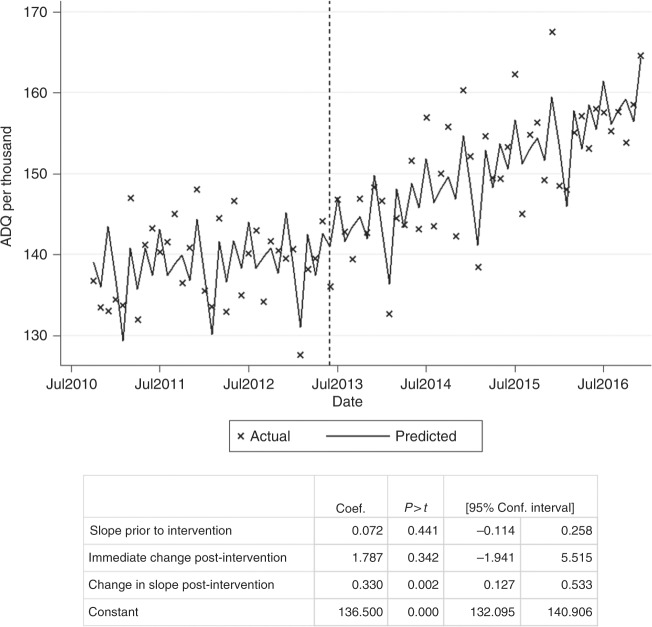
Interrupted time series analysis for total tamoxifen ADQs prescribed per 1000 population (females 35–74) per month in all practices in England

ITSA results are presented in both chart and tabular formats in Fig. 1 and indicate that between October 2010 and May 2013, the rate of tamoxifen prescribing was approximately constant, at around 137 ADQs per 1000 population per month (gradient 0.072, *p* = 0.441). Following the release of guidelines in June 2013 recommending the additional use of tamoxifen for chemoprevention, there was no immediate step change in prescribing (*p* = 0.342). However, there was a significant change in slope from 0.072 to 0.402 ADQs per 1000 population per month between June 2013 and December 2016 (+0.330, *p* = 0.001).

This change (+0.330 ADQs per 1000 population per month) equates to a total increase of 4545 ADQs per month (applied to the eligible 35–74 population of 13.8 m, December 2016), or 151 daily doses per day, i.e. 151 new patients taking tamoxifen each month. Applying this rate to the population for June 2013 to December 2016, the overall increase was 6440 ADQs per day (Appendix 4). Therefore, the change in trend could account for ~6440 more women receiving tamoxifen by December 2016.

### National variation in response to NICE guidance

**Fig. 2:**
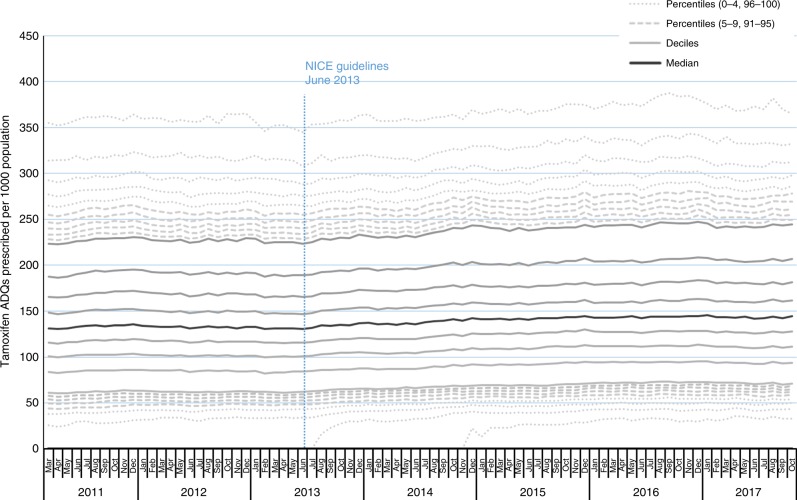
Median, deciles and extreme percentiles (0–9 and 91–100) tamoxifen ADQs prescribed per 1000 population (females 35–74) per practice across England, averaged across rolling 6-month periods

Variation in tamoxifen prescribing across all GP practices in England is presented in Fig. 2 (data set in Appendix 6), and again shows a stable rate prior to release of NICE guidelines. After this point, there is a slight increasing trend in ADQs prescribed per practice (population-adjusted) across all percentiles, which generally levels out during 2016. The second percentile became non-zero within 1 month of publication of these guidelines. Together this indicates a change across practices at all levels of prescribing.

**Fig. 3:**
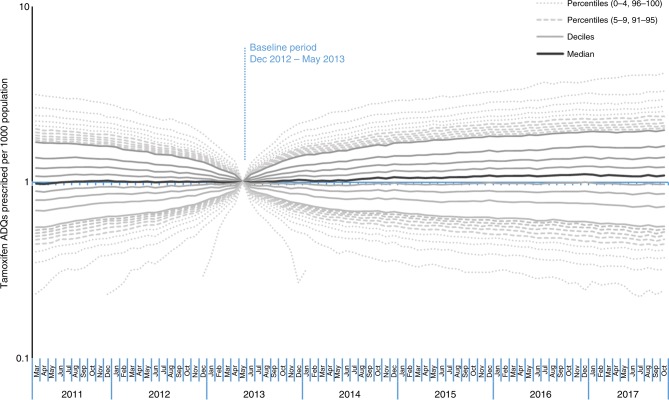
Median, deciles and extreme percentiles (0–9 and 91–100) representing fold-change in tamoxifen ADQs prescribed per 1000 population per practice across England, for rolling 6-month periods, calculated as a ratio to the baseline period. A logarithmic scale is used and zero values are excluded

Measuring the change occurring per practice, rather than overall prescribing volume, would identify whether the change in prescribing is limited to a subset of practices. Given that doctors have been reported to vary widely in their awareness of the change in guidance^7^ there may be a subset of practices responding differentially to the guidance, which would lead to asymmetry in the ratio of prescribing rates before and after guidance publication. The ratios of tamoxifen prescribing compared to the baseline period are presented as percentiles in Fig. 3. The median increases slightly over time (reaching a peak of 1.11 at July–Dec 2016) and the lowest percentiles become closer to the median. However, compared to the pattern prior to the release of the guidance, there is no divergence of the top percentiles from the median, and therefore no evidence that a subset of practices are responding differently to others.

### Validating ability to detect local changes in prescribing

A project in Manchester between May 2011 and November 2012 resulted in 136 out of 1279 pre-menopausal women deciding to take tamoxifen chemoprevention.^11^ All women aged 33–46 at moderate or high risk being regularly screened and eligible for tamoxifen chemoprevention were invited to participate. It is possible that this may have caused a local ‘saturation of the market’, restricting the opportunity for increased tamoxifen prescribing following release of the guidelines shortly afterwards. The uptake resulting from the study would be expected to cause an increase in monthly tamoxifen prescribing of 4080 ADQs (136 × 30 days), or a 5.7% increase on the average rate prior to recruitment of 71,600 ADQs/month (Dec 2010–April 2011; Appendix 4).

**Fig. 4:**
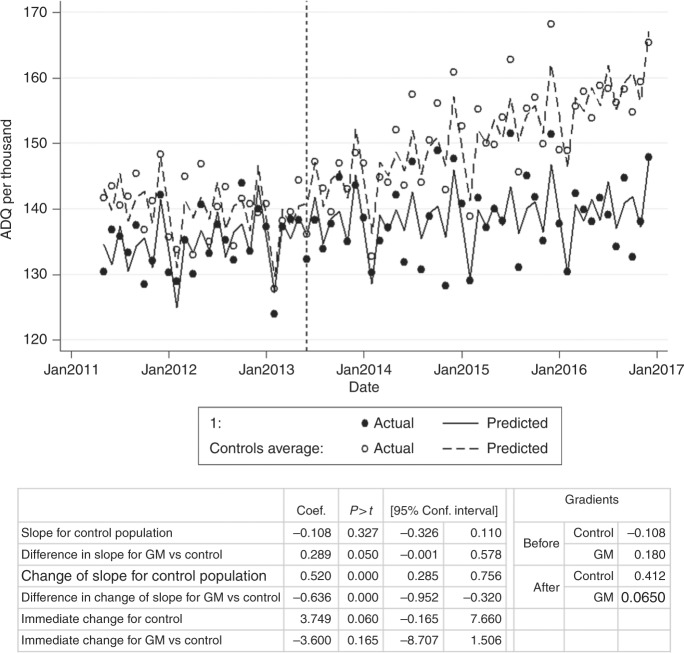
Interrupted time series analysis for total tamoxifen ADQs prescribed per 1000 population (female 35–74) per month in GM practices (filled) compared to others (control, outlines)

Time series analysis comparing tamoxifen prescribing for practices in GM (*N* = 423) against the rest of the country (control, *N* = 7659) is presented in Fig. 4. Between May 2011 (recruitment start date) and May 2013 (guideline release), the prescribing rate of the national control population showed a slight but non-significant decline (gradient −0.108, *p* = 0.327). The rate of change in GM practices was positive: 0.180 ADQs per 1000 population per month, with weak evidence that this was different to the national control population (*p* = 0.050). The average number of ADQs per day increased by 239 (2404 Dec 2010–May 2011 to 2643 Dec 2012–May 2013; Appendix 4), which would more than account for the trial participants.

Following release of the guidelines, there was a significant change in the gradient of the prescribing rate for the control population, from −0.108 to 0.412 ADQs per 1000 population per month (+0.520, *p* < 0.001, Fig. 4). In GM, however, the change of rate was negative, and significantly different (*p* < 0.001), decreasing from 0.180 to 0.0650 (−0.115). This is consistent with the opportunity for additional tamoxifen prescribing in response to NICE guidelines being lower in the Manchester area due to the prior implementation of the change during the trial.

**Fig. 5:**
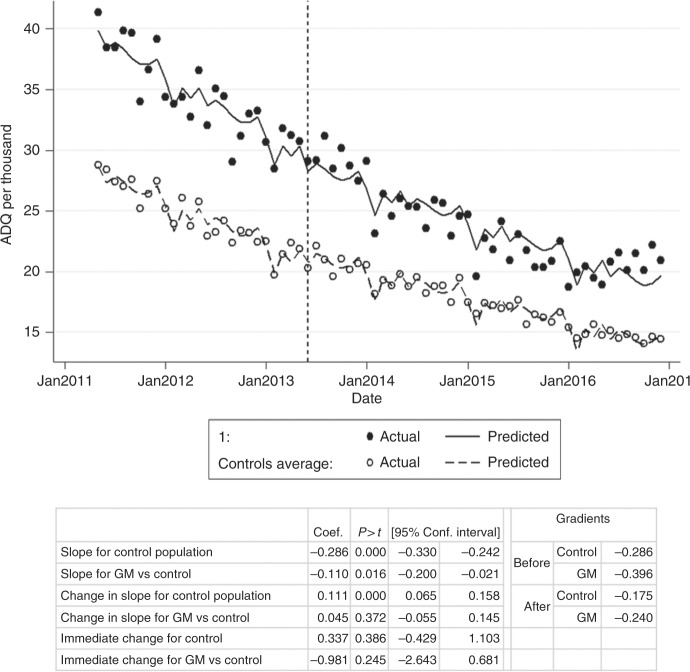
Interrupted time series analysis for total raloxifene ADQs prescribed per 1000 population (female 45 + ) per month in GM practices (‘1’, filled) compared to others (‘controls’, outlines)

Other alternatives to tamoxifen for adjuvant treatment and prevention of breast cancer are raloxifene and aromatase inhibitors; as such, their usage may interact with tamoxifen prescribing rates. Raloxifene was included in the NICE guidelines as an alternative preventative treatment to tamoxifen for post-menopausal women; but neither this drug nor patient cohort were included in the Manchester study, therefore the local change in raloxifene prescribing in response to the guidelines should not expected to be different to the national response. Raloxifene prescribing prior to NICE guidelines was much lower than that of tamoxifen and shows a decreasing trend both in GM (−0.396 ADQs per 1000 population per month) and nationally (−0.286, *p* < 0.001) (Fig. 5). Following release of guidelines, the rate of decline was slowed in the national data by 0.111 (from −0.286 to −0.175, *p* < 0.001) and in GM by 0.157 (−0.396 to −0.240), with a non-significant differential rate change between the two (*p* = 0.372). This would account for an additional 1890 new patients taking raloxifene nationally and 120 in GM up to December 2016 (Appendix 4). Anastrozole, an aromatase inhibitor, was prescribed at a slightly lower rate than tamoxifen, and prescribing was declining up to 2013 but since remained stable across most of the country throughout 2013–2017 (120–140 ADQ/month per 1000 population), but lower in GM, and continuing to decline slowly to 60–70 ADQ/month in 2017 (Figure S1a, Appendix 1). The prescribing rate of the other aromatase inhibitors combined was much higher than tamoxifen, and has been increasing nationally since 2010, approaching 200 ADQ/month per 1000 population in 2017 (300 ADQ/month in GM) (Figure S1b, Appendix 1).

### Impact of NICE guidance

We estimated above that changes in prescribing patterns between 2013 and 2016 could account for 6440 new women taking up tamoxifen and 2010 raloxifene, or 8450 in total.

Previous estimates suggest 500,000 women are eligible for chemoprevention.^12^ This is ~3.7% of England’s female population aged 35–74, the proportion of women previously found to have moderate or high risk of breast cancer by application of NICE criteria.^13^ Among those eligible, uptake in English studies has been around 10%^14,15^. Therefore, if all 500,000 women were offered tamoxifen or raloxifene following publication of the NICE guidelines, and 10% were to accept, uptake would be 50,000.

However, this will represent an overestimate, since the uptake rates were calculated after application of exclusion criteria such as pregnancy, mastectomy or recent cancer diagnosis; total numbers excluded were not reported and are difficult to estimate. For example, women at moderate risk have a 3–8% chance of breast cancer between ages 40 and 50, with whole lifetime risk 17–30%^4^. Conception rates in 2015 were 6.9% in the 35–39 age group but only 1.5% in over 40 s.^27^

Our finding of 8450 leaves a large 83% shortfall against the 50,000 estimate, unlikely to be accounted for by ineligibility. This indicates that not all eligible women were offered tamoxifen, or that uptake was lower than preceding estimates would predict. Our uptake figure does not take into account the typical dropout rate of around 40% at 5 years.^12^ However, only those persisting will have the full protective benefit.

Previous studies in this population estimate that 22 women need to take tamoxifen for one invasive breast cancer to be prevented in the first 20 years (or 45 cases prevented per 1000 women taking the drug).^6^ Offering these drugs to 500,000 women aged 35–74, in keeping with NICE guidelines, with an estimated 8% uptake (allowing for an ineligibility rate of 20%), would in principle therefore result in prevention of up to 1818 cancers (assuming complete adherence). The increase in prescribing we observed would account for 384 breast cancers prevented, leaving a shortfall of up to 1434 avoidable cancers attributable to the implementation of the NICE guideline falling short of what would be predicted.

## Discussion

### Summary

Prior to release of NICE guidelines recommending tamoxifen for chemoprevention, time series analysis indicates that its rate of prescribing was approximately constant. The release of a new guideline in June 2013 markedly increasing the number of women eligible for treatment was followed by a trend to increased prescribing, accounting for ~6437 more women being prescribed tamoxifen by December 2016, but no step change. The increase appeared to level off after 2016. We found no evidence for the existence of any sub-group of practices responding differently to the national trend. However, the increase in prescribing of tamoxifen was slower for practices located in GM, perhaps due to a preceding intervention which increased use of tamoxifen for chemoprevention prior to the NICE guidelines. Prescribing of raloxifene, recommended within the guideline as an alternative for post-menopausal women, has been generally declining across the country with a slight decrease in its rate of decline following release of the NICE guidelines; there was no evidence that the rate of change in GM was any different.

### Strengths and limitations

A key strength of our analysis is that it covers the complete data for all prescription items dispensed in England, not a sample. There is no national data set of patient-level data, but were such a data set to be available, it would allow a more detailed evaluation. For example, it would permit more accurate counting of the number of distinct recipients (although the ADQs used in this study are likely to be a reliable proxy for tamoxifen users). It would also allow us to limit our analysis to only those without existing cancer diagnoses, where usage is preventive in line with new guidelines (however this new preventive patient group is so much larger than the previous diagnosed group eligible for treatment that the analysis conducted is sufficiently robust to detect a change in prescribing behaviour). We were unable to measure prescribing in secondary or tertiary care, meaning initial prescriptions issued by a hospital clinic and prescriptions dispensed in hospital are not included in the data; however, prescribing of tamoxifen is typically taken over by GPs in the NHS.^16^

The gradual increase in prescribing we report accounts for ~6440 additional tamoxifen and 2010 raloxifene recipients (8450 in total) potentially attributable to NICE’s impact. This may be an underestimate of the number of new recipients due to the typical dropout rate of around 40% at 5 years.^12^ However, only those persisting will have the full protective benefit. No routinely collected data can accurately measure adherence to therapy, but the fact that a prescription was written and, in addition, dispensed indicates that there was agreement from the patient to take the medication, which is what we were seeking to measure.

Although we did not detect a statistically significant increase in tamoxifen prescribing in GM during study recruitment, we would not have expected to do so, given the small number of participants relative to background tamoxifen prescribing. After guideline release, the differential change of rate in tamoxifen prescribing identified in GM could be due to a saturation of the market effect from this study, but any local policy restrictions on implementation of the new guidelines could also contribute to a slower uptake in this area. The higher prescribing rates of raloxifene and two of the aromatase inhibitors in this area could also have contributed to the slow response, by limiting the number of post-menopausal women eligible to take tamoxifen preventively. Anastrozole usage was lower in GM than other practices, and has not been increasing despite a major study reporting the effectiveness of anastrozole in breast cancer prevention in 2014^17^ and its addition to NICE guidelines in March 2017.

As we were able to adjust for seasonal variation, the ITSA method used here otherwise assumes that trends are linear and unaffected by any stimuli other than the intervention under investigation.^10^ Regarding other potential sources of change in usage: there were some media reports on tamoxifen during the study period but no drug safety alerts for either drug on the UK government website; prices for some generic formulations of tamoxifen have increased between 2011 and 2016, but not for the standard 20 mg tablets^18^; and NICE guidelines for tamoxifen have not changed substantially. The general decline in raloxifene is likely due to other treatments being recommended ahead of it for its main indication, osteoporosis. Variations in prescribing of tamoxifen are also accountable to breast cancer rates and uptake amongst cancer patients. However, although incidence of breast cancer diagnosis is increasing over time,^1^ it seems unlikely that the change in slope of tamoxifen prescribing observed in 2013 could be explained by this. Guidelines have not changed substantially with respect to tamoxifen treatment for either local or advanced breast cancer since 2009.^2,3^ We did not see any substantial shifts in prescribing trends for other adjuvant/preventative breast cancer drugs.

### Findings in context of other research

We found no evidence for a step change in prescribing, despite the guideline causing a large and immediate increase in the population appropriate for tamoxifen. Long lead times to first prescription may have contributed to the slow adoption rate, due to referral to specialist clinics, the complex decision process for patients^19^ and the eligible population being healthy and therefore not visiting their GP frequently or actively seeking therapy. Furthermore, a large increase may not be expected, given that patient acceptance of chemoprevention can be low for a multitude of reasons.^12^ Uptake during trials and in highly specialist clinics has been around 10%,^14,15^ but uptake in routine practice is unknown.

We attempted to place our results on the impact of NICE guidance in the context of what is achievable in chemoprevention. Our finding of 8450 women taking up chemoprevention leaves a large 83% shortfall against the 50,000 estimated uptake from complete coverage, unlikely to be accounted for by ineligibility. This may indicate that not all eligible women were offered tamoxifen, or that uptake was lower than preceding estimates would predict.

A NICE economic impact assessment estimated a reduction of 11 cancers over 50 years per 1000 women offered tamoxifen or raloxifene.^20^ However, setting our findings in the context of the NICE forecast is challenging. Contrary to current literature, which gives a 10% estimate for uptake^14,15^, NICE assumed an uptake rate of 25% (with no source given, and scenarios additionally modelled with 50 and 75% uptake); and a 50% one-year dropout rate (attributed to ‘expert opinion’). In estimating 50-year risks, they used a risk reduction figure of 35% averaged from two studies with only up to 10 years follow-up data, and applied this to unpublished annual cancer incidence rates obtained from personal communication. The potential total eligible population and uptake were not calculated.

Consistent with our results, previous studies find that the response in primary care to new guidelines generally falls short of expectations.^21–26^ Sometimes this may be because a change in practice precedes the guidelines; however the initial steady rate of tamoxifen prescribing we report follows a period of decline between 2001 and 2009.^26^

An extensively reported GP survey showed wide variation in GPs’ knowledge of change in the evidence and guidance on preventive treatment.^7^ Our results do not indicate that the change in use of tamoxifen was concentrated in a subset of practices. This lack of variation in response by individual practices may be because chemoprevention is often discussed in specialist clinics, each of which will cover a range of practices. However, this discussion will often be initiated in primary care,^8^ thus leaving room for variation in care driven by variation in primary care physicians’ knowledge.

### Policy implications and future research

We found evidence, consistent with previous work, that changes in practice warranted by new guidelines and evidence are implemented slowly and perhaps incompletely. This is testament to the challenge in disseminating knowledge effectively. This work was conducted as part of our OpenPrescribing.net project, an openly accessible data service which highlights prescribing variation in primary care, and allow practices and commissioners to monitor their own prescribing behaviour for key prescribing measures and any chemical of interest, using statistical process control techniques to automatically send alerts to practices when they deviate from national changes in behaviour. We suggest that greater investment in disseminating evidence, auditing its implementation, and using variation in practice to target clinicians for educational interventions may all prove to be cost effective mechanisms to ensure that health services can realise the value of public investment on both primary research and on generating guidelines. The latest data on tamoxifen prescribing for each of England's practices and CCGs can be viewed online as part of our live prescribing data explorer, at openprescribing.net/researchmeasures.

## Conclusions

Following release of guidelines advising use of tamoxifen for chemoprevention in a new larger cohort of women, we found a gradual increase in the rate of tamoxifen prescribing estimated to account for 8450 patients over 2.5 years. Although difficult to estimate exclusion rates, this represents only 17% of a feasible uptake overall, even after accounting for 90% of patients refusing an offer of chemoprevention. We did not find evidence suggestive of the response being concentrated in certain practices, and verified that local variation can be identified in our data set. Overall, our results suggest not all women at increased risk of breast cancer have been offered tamoxifen chemoprevention, potentially leaving them exposed to avoidable risk. Improving dissemination of guidance to healthcare professionals and routinely monitoring its implementation could help to reduce this risk.

## Electronic supplementary material

Appendices 1-3(PDF 185 kb)

Appendix 4(XLSX 97 kb)

Appendix 5(XLSX 13 kb)

Appendix 6(XLSX 202 kb)

Supplementary materials
