# Supplementary material for: Impact of NICE guidance on tamoxifen prescribing in England 2011–2017: an interrupted time series analysis
Source: Br J Cancer. 2018 Apr 23;118(9):1268–75. doi: 10.1038/s41416-018-0065-2 (PMC5943266; doi:10.1038/s41416-018-0065-2)
Supplement: Supplementary file 1 — Appendices 1-3(PDF 185 kb) [file 41416_2018_65_MOESM1_ESM.pdf]

Impact of NICE Guidance on Tamoxifen Prescribing in England 2011-2017 - An Interrupted Time Series Analysis

Supplementary Material

Appendix 1. Additional Tables and Figures

Table S1. BNF Codes, Names and dosages for tamoxifen, raloxifene and anastrozole. Dosage indicates factor by which Quantity was divided to calculate Average Daily Quantity (ADQ).

Chemical	BNF Code	BNF Name	Dosage
Tamoxifen	0803041S0AAAAAA	Nolvadex D_Tab 20mg	1
	0803041S0AAABAB	Nolvadex_Tab 10mg	2
	0803041S0AAACAC	Soltamox_Oral Soln 10mg/5ml S/F	10
	0803041S0AAAHAH	Tamoxifen Cit_Liq Spec 10mg/5ml	10
		Tamoxifen Cit_Oral Soln 10mg/5ml S/F	10
	0803041S0AAAJAJ	Tamoxifen Cit_Oral Susp 10mg/5ml	10
	0803041S0BCAAAA	Tamoxifen Cit_Tab 10mg	2
	0803041S0BCABAB	Tamoxifen Cit_Tab 20mg	1
	0803041S0BLAAAJ	Tamoxifen Cit_Tab 40mg	0.5
Raloxifene	0604011X0AAAAAA	Raloxifene HCl_Tab 60mg	1
	0604011X0BBAAAA	Evista_Tab 60mg	1
	0604011X0BDAAAA	Razylan_Tab 60mg	1
	0604011X0BEAAAA	Ostiral_Tab 60mg	1
	0604011X0BFAAAA	Evirex_Tab 60mg	1
Anastrozole (generic from June 2017)	0803041B0AAAAAA	Anastrozole_Tab 1mg	1
	0803041B0AAABAB	Anastrozole_Liq Spec 1mg/5ml	5
	0803041B0AAACAC	Anastrozole_Cap 1mg	1
	0803041B0BBAAAA	Arimidex_Tab 1mg	1
	0803041B0BCAAAA	Nastrosa_Tab 1mg	1
Letrozole (generic from Jan 2012)	0803041L0AAAAAA	Letrozole_Tab 2.5mg	1
	0803041L0BBAAAA	Femara_Tab 2.5mg	1
Exemestane (generic from Jan 2012)	0803041C0AAAAAA	Exemestane_Tab 25mg	1
	0803041C0BBAAAA	Aromasin_Tab 25mg	1

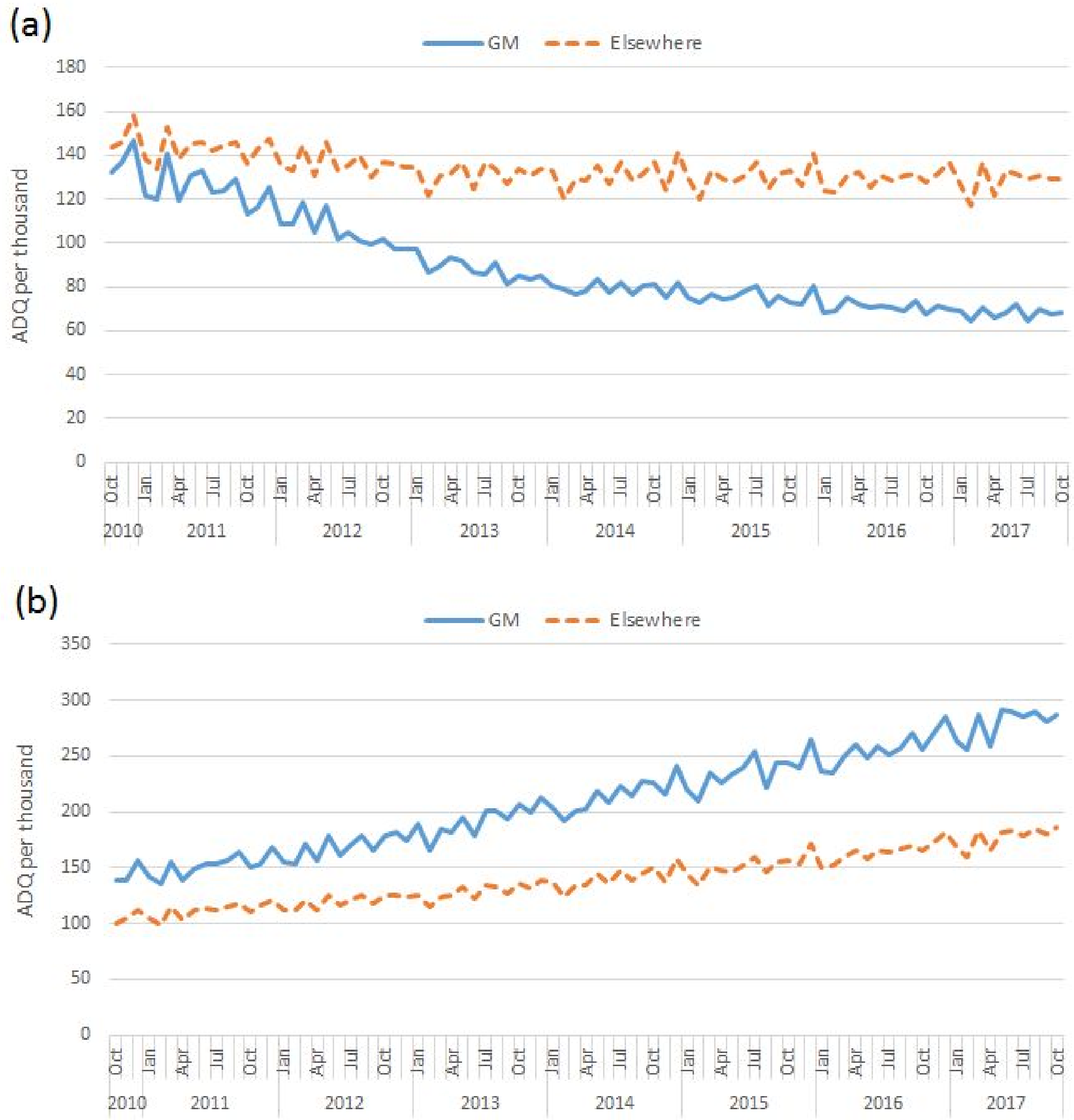

Figure S1. Total ADQs dispensed per 1,000 population (females 45+) in Greater Manchester practices versus all other practices in England for (a) anastrozole and (b) exemestane and letrozole.

Appendix 2. SQL Codes for data extraction

1. Extract all prescribing data into new tables

a. ebmdatalab.helen.tamoxifen_all_prescribing_20180102

```
SELECT *
      FROM ebmdatalab.hscic.normalised_prescribing_standard p
      WHERE SUBSTR(bnf_code,1,9) = '0803041S0'
```

b. ebmdatalab.helen.raloxifene_all_prescribing_20180102

```
SELECT *
      FROM ebmdatalab.hscic.normalised_prescribing_standard p
      WHERE SUBSTR(bnf_code,1,9) = '0604011X0'
```

c. ebmdatalab.helen.anastrozole_all_prescribing

```
SELECT *
      FROM ebmdatalab.hscic.normalised_prescribing_standard p
      WHERE SUBSTR(bnf_code,1,9) = '0604011X0'
```

d. ebmdatalab.helen.exemestane_letrozole_all_prescribing

```
SELECT *
      FROM ebmdatalab.hscic.normalised_prescribing_standard p
      WHERE SUBSTR(bnf_code,1,9) IN ('0803041C0',’0803041L0’)
```

2. Produce aggregated prescribing data to determine when each practice was active. Save to ebmdatalab.aggregated_data.prescribing_by_mo_by_practice_all_years

```
SELECT
    SUBSTR(bnf_code,1,2) AS Chapter,
    practice,
    EXTRACT(year FROM month) AS year,
    EXTRACT(month FROM month) AS month,
    CAST(month AS DATE) AS year_mon,
    sum(items) AS items,
    sum(quantity) AS quantity
FROM ebmdatalab.hscic.normalised_prescribing_standard p
GROUP BY
    Chapter,
    practice,
    year,
    month,
    year_mon
```

3. Tamoxifen prescribing by practice, averaged over 6 month periods (for percentiles)

```
WITH
manc AS (
SELECT DISTINCT code, SUBSTR(POSTCODE,1,3) AS POSTCODE_SECTOR

FROM ebmdatalab.hscic.practices
```

```
WHERE SUBSTR(POSTCODE,1,3) IN
('BL1','BL2','BL3','BL4','BL5','BL8','BL9','M1','M12','M13','M17','M18','
M19','M2','M20','M21','M22','M23','M24','M25','M26','M27','M28','M29','M3
','M30','M31','M32','M33','M34','M35','M38','M41','M43','M44','M45','M46'
,'M5','M60','M7','M8','M9','OL1','OL2','OL3','OL4','OL5','OL6','OL7','OL8
','OL9','SK1','SK2','SK4','SK5','SK6','SK7','SK8','WN1','WN2','WN3','WN4'
,'WN7')
OR SUBSTR(POSTCODE,1,4) IN
('OL10','OL11','OL15','OL16','SK14','SK15','SK16','SK22','WA14')
--ORDER BY POSTCODE_SECTOR
),

-- create a table for all practices for all months they exist and
prescribed more than zero total items.
-- inner join to practice list to filter for type 4 practices.
q2 AS ( SELECT
          p.practice,
          p.year_mon,
          sum(p.quantity) as quantity,
          max(s.female_35_44) + max(s.female_45_54) + max(s.female_55_64)
+ max(s.female_65_74) AS female_total,
          max(s.female_35_44)  AS female_35_44
        FROM ebmdatalab.hscic.practice_statistics s
        LEFT JOIN
ebmdatalab.aggregated_data.prescribing_by_mo_by_practice_all_years p
          ON  p.practice = s.practice AND p.year_mon = CAST(s.month AS
DATE)
        INNER JOIN  ebmdatalab.hscic.practices prac ON prac.code
=p.practice AND prac.setting = 4
        GROUP BY practice, year_mon ),

-- select all tamoxifen prescribing data, and adjust for ADQ of 20mg
(liquids are all 10mg/5ml)
t AS (SELECT practice, bnf_name, items, quantity, month,
        CASE WHEN bnf_name LIKE '%Oral%' THEN 10
             WHEN bnf_name LIKE '%Liq%' THEN 10
             WHEN RTRIM(bnf_name) LIKE '%10mg' THEN 2
             WHEN RTRIM(bnf_name) LIKE '%40mg' THEN 0.5
        ELSE 1
        END AS dosage,
        quantity/ CASE WHEN bnf_name LIKE '%Oral%' THEN 10
             WHEN bnf_name LIKE '%Liq%' THEN 10
             WHEN RTRIM(bnf_name) LIKE '%10mg' THEN 2
             WHEN RTRIM(bnf_name) LIKE '%40mg' THEN 0.5
             ELSE 1
             END AS adq
      FROM ebmdatalab.helen.tamoxifen_all_prescribing_20180102 ),
```

```
-- total tamoxifen ADQs and ADQs per 1000 females per practice per month
p AS (SELECT
        q2.practice,
        CAST(q2.year_mon AS DATE) AS month,
        SUM(t.adq) AS adq,
        MAX(q2.female_total) AS female_total,
        MAX(MAX(q2.female_35_44)) OVER (PARTITION BY q2.practice) AS
female_35_44_max,
        1000*IEEE_DIVIDE(sum(t.adq),max(q2.female_total)) AS adq_per_thou
      FROM q2
      LEFT JOIN t ON t.practice = q2.practice AND CAST(t.month AS DATE) =
q2.year_mon
      GROUP BY practice, month ),

-- join table to itself to calculate all 6-mo rolling averages of
tamoxifen prescribing as a proportion of population
-- the base table p gives us a data point for each month and the joined
table q gives a sum of the previous 6 months for each p.month
rolling AS (
      SELECT
        p.practice,
        p.month,
        SUM(q.adq) AS adq_6mo,
        AVG(q.female_total) AS avg_females_6mo,
        AVG(q.adq_per_thou) AS adq_per_thou_6mo
      FROM  p
      LEFT JOIN p AS q ON p.practice = q.practice AND q.month BETWEEN
DATE_SUB(p.month, INTERVAL 5 month) AND p.month

      WHERE p.month BETWEEN '2011-03-01' AND '2017-10-01' -- exclude
first 5 months of data such that each 6-month rolling period is complete.
        AND p.female_35_44_max > 10

      GROUP BY practice, month)


-- results including ratio calculations
SELECT r.practice,
       CASE WHEN m.code IS NOT NULL THEN 1 ELSE 0 END AS manc_flag,
       r.month,
       r.avg_females_6mo,
       r.adq_6mo,
       r.adq_per_thou_6mo,
       IEEE_DIVIDE(r.adq_per_thou_6mo,b.adq_per_thou_6mo) AS ADQ_Ratio,
       b.adq_per_thou_6mo IS NOT NULL AS NonZero_baseline
FROM rolling r
LEFT JOIN rolling b ON r.practice = b.practice and b.month = '2013-05-01'
LEFT JOIN manc m ON  m.code = r.practice
```

```
ORDER BY practice, month
```

4. Changes in tamoxifen prescribing for ITSA - all practices

```
WITH
-- create a table for all practices for all months they exist and prescribed
more than zero items.
-- inner join to practice list to filter for type 4 practices.
q2 AS (
  SELECT p.practice,
    p.year_mon, sum(p.items) as items, sum(p.quantity) as quantity,
    max(s.female_35_44) + max(s.female_45_54) + max(s.female_55_64) +
max(s.female_65_74) AS female_total,
    max(s.female_35_44)  AS female_35_44

  FROM ebmdatalab.hscic.practice_statistics s
  LEFT JOIN ebmdatalab.aggregated_data.prescribing_by_mo_by_practice_all_years
p ON  p.practice = s.practice AND p.year_mon = CAST(s.month AS DATE)
  INNER JOIN  ebmdatalab.hscic.practices prac ON prac.code =p.practice AND
prac.setting = 4

  GROUP BY practice, year_mon ),

-- select all tamoxifen prescribing data, and adjust for ADQ of 20mg (liquids
are all 10mg/5ml)
t AS (SELECT practice, bnf_name, items, quantity, month,
        CASE WHEN bnf_name LIKE '%Oral%' THEN 10
             WHEN bnf_name LIKE '%Liq%' THEN 10
             WHEN RTRIM(bnf_name) LIKE '%10mg' THEN 2
             WHEN RTRIM(bnf_name) LIKE '%40mg' THEN 0.5
        ELSE 1
        END AS dosage,
        quantity/ CASE WHEN bnf_name LIKE '%Oral%' THEN 10
             WHEN bnf_name LIKE '%Liq%' THEN 10
             WHEN RTRIM(bnf_name) LIKE '%10mg' THEN 2
             WHEN RTRIM(bnf_name) LIKE '%40mg' THEN 0.5
             ELSE 1
             END AS adq
      FROM ebmdatalab.helen.tamoxifen_all_prescribing_20180102 ),

-- total tamoxifen ADQs and ADQs per 1000 females per practice per month
p AS (SELECT
        q2.practice,
        CAST(q2.year_mon AS DATE) AS month,
        SUM(t.adq) AS adq,
        MAX(q2.female_total) AS female_total,
        MAX(MAX(q2.female_35_44)) OVER (PARTITION BY q2.practice) AS
female_35_44_max,
```

```
        1000*IEEE_DIVIDE(sum(t.adq),max(q2.female_total)) AS adq_per_thou
      FROM q2
      LEFT JOIN t ON t.practice = q2.practice AND CAST(t.month AS DATE) =
q2.year_mon
      GROUP BY  practice, month ),

-- Group all practices
p2 AS (
    SELECT
        p.month,
        SUM(p.adq) AS adq,
        SUM(p.female_total) AS female_total,
        1000*IEEE_DIVIDE(sum(p.adq),sum(p.female_total)) AS adq_perthou
    FROM  p
    WHERE p.month BETWEEN '2010-10-01' AND '2017-10-01' -- include Oct-2011
onward such that we include same periods as the 6-month rolling data.
      AND p.female_35_44_max > 10
    GROUP BY month )

-- results including ratio calculations
SELECT
    p2.month,
    p2.adq,
    p2.female_total,
    p2.adq_perthou,
    IEEE_DIVIDE(p2.adq_perthou,b.adq_perthou) AS ADQ_Ratio
FROM p2, p2 AS b WHERE b.month = '2013-05-01'
ORDER BY month
```

5. Changes in tamoxifen prescribing for ITSA - flagging greater Manchester prescribing, to search for changes in response to clinical trial.

```
WITH
manc AS (
SELECT DISTINCT code, SUBSTR(POSTCODE,1,3) AS POSTCODE_SECTOR
FROM ebmdatalab.hscic.practices
WHERE SUBSTR(POSTCODE,1,3) IN
('BL1','BL2','BL3','BL4','BL5','BL8','BL9','M1','M12','M13','M17','M18','M19','
M2','M20','M21','M22','M23','M24','M25','M26','M27','M28','M29','M3','M30','M31
','M32','M33','M34','M35','M38','M41','M43','M44','M45','M46','M5','M60','M7','
M8','M9','OL1','OL2','OL3','OL4','OL5','OL6','OL7','OL8','OL9','SK1','SK2','SK4
','SK5','SK6','SK7','SK8','WN1','WN2','WN3','WN4','WN7')
OR SUBSTR(POSTCODE,1,4) IN
('OL10','OL11','OL15','OL16','SK14','SK15','SK16','SK22','WA14') ),
```

```
-- create a table for all practices for all months they exist and prescribed more than zero items.
-- inner join to practice list to filter for type 4 practices.
q2 AS (
  SELECT p.practice,
    p.year_mon, sum(p.items) as items, sum(p.quantity) as quantity,
    max(s.female_35_44) + max(s.female_45_54) + max(s.female_55_64) + max(s.female_65_74) AS female_total,
    max(s.female_35_44)  AS female_35_44

  FROM ebmdatalab.hscic.practice_statistics s
  LEFT JOIN ebmdatalab.aggregated_data.prescribing_by_mo_by_practice_all_years p ON  p.practice = s.practice AND p.year_mon = CAST(s.month AS DATE)
  INNER JOIN  ebmdatalab.hscic.practices prac ON prac.code =p.practice AND prac.setting = 4

  GROUP BY practice, year_mon ),

-- select all tamoxifen prescribing data, and adjust for ADQ of 20mg (liquids are all 10mg/5ml)
t AS (SELECT practice, bnf_name, items, quantity, month,
        CASE WHEN bnf_name LIKE '%Oral%' THEN 10
             WHEN bnf_name LIKE '%Liq%' THEN 10
             WHEN RTRIM(bnf_name) LIKE '%10mg' THEN 2
             WHEN RTRIM(bnf_name) LIKE '%40mg' THEN 0.5
        ELSE 1
        END AS dosage,
        quantity/ CASE WHEN bnf_name LIKE '%Oral%' THEN 10
             WHEN bnf_name LIKE '%Liq%' THEN 10
             WHEN RTRIM(bnf_name) LIKE '%10mg' THEN 2
             WHEN RTRIM(bnf_name) LIKE '%40mg' THEN 0.5
             ELSE 1
             END AS adq
      FROM ebmdatalab.helen.tamoxifen_all_prescribing_20180102 ),


-- total tamoxifen ADQs and ADQs per 1000 females per practice per month
p AS (SELECT
        CASE WHEN m.code IS NOT NULL THEN 1 ELSE 0 END AS manc_flag,
        q2.practice,
        CAST(q2.year_mon AS DATE) AS month,
        SUM(t.adq) AS adq,
        MAX(q2.female_total) AS female_total,
        MAX(MAX(q2.female_35_44)) OVER (PARTITION BY q2.practice) AS female_35_44_max,
        1000*IEEE_DIVIDE(sum(t.adq),max(q2.female_total)) AS adq_per_thou
      FROM q2
```

```
      LEFT JOIN t ON t.practice = q2.practice AND CAST(t.month AS DATE) =
q2.year_mon
      LEFT JOIN manc m ON  m.code = q2.practice
      GROUP BY  practice, month, manc_flag),


-- Group practices up to show only -/+ Manchester flag
p2 AS (
      SELECT  p.manc_flag,
              p.month,
              SUM(p.adq) AS adq,
              SUM(p.female_total) AS female_total,
              1000*IEEE_DIVIDE(sum(p.adq),sum(p.female_total)) AS adq_perthou
      FROM  p
      WHERE p.month BETWEEN '2010-12-01' AND '2017-10-01'
        AND p.female_35_44_max > 10
      GROUP BY manc_flag, month )

-- results including ratio calculations
SELECT
    p2.manc_flag,
    p2.month,
    p2.adq,
    p2.female_total,
    p2.adq_perthou,
    IEEE_DIVIDE(p2.adq_perthou,b.adq_perthou) AS ADQ_Ratio
FROM p2
LEFT JOIN p2 AS b ON b.manc_flag = p2.manc_flag AND b.month = '2013-05-01'
ORDER BY month, manc_flag
```

6. Changes in raloxifene prescribing for ITSA - flagging greater Manchester prescribing.

```
WITH
manc AS (
SELECT DISTINCT code, SUBSTR(POSTCODE,1,3) AS POSTCODE_SECTOR
FROM ebmdatalab.hscic.practices
WHERE SUBSTR(POSTCODE,1,3) IN
('BL1','BL2','BL3','BL4','BL5','BL8','BL9','M1','M12','M13','M17','M18','M19','
M2','M20','M21','M22','M23','M24','M25','M26','M27','M28','M29','M3','M30','M31
','M32','M33','M34','M35','M38','M41','M43','M44','M45','M46','M5','M60','M7','
M8','M9','OL1','OL2','OL3','OL4','OL5','OL6','OL7','OL8','OL9','SK1','SK2','SK4
','SK5','SK6','SK7','SK8','WN1','WN2','WN3','WN4','WN7')
OR SUBSTR(POSTCODE,1,4) IN
('OL10','OL11','OL15','OL16','SK14','SK15','SK16','SK22','WA14') ),

-- create a table for all practices for all months they exist and prescribed
more than zero items.
```

```
-- inner join to practice list to filter for type 4 practices.
q2 AS (
  SELECT p.practice,
    p.year_mon, sum(p.items) as items, sum(p.quantity) as quantity,
    max(s.female_45_54) + max(s.female_55_64) + max(s.female_65_74) +
max(female_75_plus) AS female_total,
    -- use post-menopausal population only for raloxifene
    max(s.female_35_44)  AS female_35_44

  FROM ebmdatalab.hscic.practice_statistics s
  LEFT JOIN ebmdatalab.aggregated_data.prescribing_by_mo_by_practice_all_years
p ON  p.practice = s.practice AND p.year_mon = CAST(s.month AS DATE)
  INNER JOIN  ebmdatalab.hscic.practices prac ON prac.code =p.practice AND
prac.setting = 4

  GROUP BY practice, year_mon ),

-- select all **raloxifene** prescribing data, no adjustment required, all are
60mg tablets, as at Oct 2017.

t AS (SELECT practice, bnf_name, items, quantity, month,
       1 AS dosage,
       quantity/ 1 AS adq
      FROM ebmdatalab.helen.raloxifene_all_prescribing_20180102 p
      order by practice, month ),


-- total raloxifene ADQs and ADQs per 1000 females per practice per month
p AS (SELECT
        CASE WHEN m.code IS NOT NULL THEN 1 ELSE 0 END AS manc_flag,
        q2.practice,
        CAST(q2.year_mon AS DATE) AS month,
        SUM(t.adq) AS adq,
        MAX(q2.female_total) AS female_total,
        MAX(MAX(q2.female_35_44)) OVER (PARTITION BY q2.practice) AS
female_35_44_max,
        1000*IEEE_DIVIDE(sum(t.adq),max(q2.female_total)) AS adq_per_thou
      FROM q2
      LEFT JOIN t ON t.practice = q2.practice AND CAST(t.month AS DATE) =
q2.year_mon
      LEFT JOIN manc m ON  m.code = q2.practice
      GROUP BY  practice, month, manc_flag),


-- Group practices up to show only -/+ Manchester flag
p2 AS (
      SELECT  p.manc_flag,
              p.month,
```

```
            SUM(p.adq) AS adq,
            SUM(p.female_total) AS female_total,
            1000*IEEE_DIVIDE(sum(p.adq),sum(p.female_total)) AS adq_perthou
    FROM  p
    WHERE p.month BETWEEN '2010-12-01' AND '2017-10-01'
      AND p.female_35_44_max > 10
    GROUP BY manc_flag, month )

-- results including ratio calculations
SELECT
    p2.manc_flag,
    p2.month,
    p2.adq,
    p2.female_total,
    p2.adq_perthou,
    IEEE_DIVIDE(p2.adq_perthou,b.adq_perthou) AS ADQ_Ratio
FROM p2
LEFT JOIN p2 AS b ON b.manc_flag = p2.manc_flag AND b.month = '2013-05-01'
ORDER BY month, manc_flag
```

7. Changes in anastrozole prescribing for chart.

```
-- anastrozole manc vs elsewhere
WITH
manc AS (
SELECT DISTINCT code, SUBSTR(POSTCODE,1,3) AS POSTCODE_SECTOR
FROM ebmdatalab.hscic.practices
WHERE SUBSTR(POSTCODE,1,3) IN
('BL1','BL2','BL3','BL4','BL5','BL8','BL9','M1','M12','M13','M17','M18','M19','
M2','M20','M21','M22','M23','M24','M25','M26','M27','M28','M29','M3','M30','M31
','M32','M33','M34','M35','M38','M41','M43','M44','M45','M46','M5','M60','M7','
M8','M9','OL1','OL2','OL3','OL4','OL5','OL6','OL7','OL8','OL9','SK1','SK2','SK4
','SK5','SK6','SK7','SK8','WN1','WN2','WN3','WN4','WN7')
OR SUBSTR(POSTCODE,1,4) IN
('OL10','OL11','OL15','OL16','SK14','SK15','SK16','SK22','WA14') ),

-- create a table for all practices for all months they exist and prescribed
more than zero items.
-- inner join to practice list to filter for type 4 practices.
q2 AS (
  SELECT p.practice,
    p.year_mon, sum(p.items) as items, sum(p.quantity) as quantity,
    max(s.female_45_54) + max(s.female_55_64) + max(s.female_65_74) +
max(female_75_plus) AS female_total,
    max(s.female_35_44)  AS female_35_44

  FROM ebmdatalab.hscic.practice_statistics s
```

```
  LEFT JOIN ebmdatalab.aggregated_data.prescribing_by_mo_by_practice_all_years
p ON  p.practice = s.practice AND p.year_mon = CAST(s.month AS DATE)
  INNER JOIN  ebmdatalab.hscic.practices prac ON prac.code =p.practice AND
prac.setting = 4

  GROUP BY practice, year_mon ),

-- select all anastrozole prescribing data, and adjust for ADQ of 1mg (liquids
are 1mg/5ml)
t AS (SELECT practice, bnf_name, items, quantity, month,
        CASE WHEN bnf_name LIKE '%Oral%' THEN 5
             WHEN bnf_name LIKE '%Liq%' THEN 5
        ELSE 1
        END AS dosage,
        quantity/ CASE WHEN bnf_name LIKE '%Oral%' THEN 5
             WHEN bnf_name LIKE '%Liq%' THEN 5
             ELSE 1
             END AS adq
      FROM ebmdatalab.helen.anastrozole_all_prescribing ),


-- total anastrozole ADQs and ADQs per 1000 females per practice per month
p AS (SELECT
        CASE WHEN m.code IS NOT NULL THEN 1 ELSE 0 END AS manc_flag,
        q2.practice,
        CAST(q2.year_mon AS DATE) AS month,
        SUM(t.adq) AS adq,
        MAX(q2.female_total) AS female_total,
        MAX(MAX(q2.female_35_44)) OVER (PARTITION BY q2.practice) AS
female_35_44_max,
        1000*IEEE_DIVIDE(sum(t.adq),max(q2.female_total)) AS adq_per_thou
      FROM q2
      LEFT JOIN t ON t.practice = q2.practice AND CAST(t.month AS DATE) =
q2.year_mon
      LEFT JOIN manc m ON  m.code = q2.practice
      GROUP BY  practice, month, manc_flag),


-- Group practices up to show only -/+ Manchester flag
p2 AS (
      SELECT  p.manc_flag,
              p.month,
              SUM(p.adq) AS adq,
              SUM(p.female_total) AS female_total,
              1000*IEEE_DIVIDE(sum(p.adq),sum(p.female_total)) AS adq_perthou
      FROM  p
      WHERE p.month BETWEEN '2010-10-01' AND '2017-10-01'
        AND p.female_35_44_max > 10
```

```
      GROUP BY manc_flag, month )

-- results including ratio calculations
SELECT
    p2.manc_flag,
    p2.month,
    p2.adq,
    p2.female_total,
    p2.adq_perthou
FROM p2
ORDER BY month, manc_flag
```

8. Changes in exemestane and letrozole prescribing for chart.

```
-- exemestane and letrozole manc vs elsewhere
WITH
manc AS (
SELECT DISTINCT code, SUBSTR(POSTCODE,1,3) AS POSTCODE_SECTOR
FROM ebmdatalab.hscic.practices
WHERE SUBSTR(POSTCODE,1,3) IN
('BL1','BL2','BL3','BL4','BL5','BL8','BL9','M1','M12','M13','M17','M18','M19','
M2','M20','M21','M22','M23','M24','M25','M26','M27','M28','M29','M3','M30','M31
','M32','M33','M34','M35','M38','M41','M43','M44','M45','M46','M5','M60','M7','
M8','M9','OL1','OL2','OL3','OL4','OL5','OL6','OL7','OL8','OL9','SK1','SK2','SK4
','SK5','SK6','SK7','SK8','WN1','WN2','WN3','WN4','WN7')
OR SUBSTR(POSTCODE,1,4) IN
('OL10','OL11','OL15','OL16','SK14','SK15','SK16','SK22','WA14') ),

-- create a table for all practices for all months they exist and prescribed
more than zero items.
-- inner join to practice list to filter for type 4 practices.
q2 AS (
  SELECT p.practice,
    p.year_mon, sum(p.items) as items, sum(p.quantity) as quantity,
    max(s.female_45_54) + max(s.female_55_64) + max(s.female_65_74) +
max(female_75_plus) AS female_total,
    max(s.female_35_44)  AS female_35_44

  FROM ebmdatalab.hscic.practice_statistics s
  LEFT JOIN ebmdatalab.aggregated_data.prescribing_by_mo_by_practice_all_years
p ON  p.practice = s.practice AND p.year_mon = CAST(s.month AS DATE)
  INNER JOIN  ebmdatalab.hscic.practices prac ON prac.code =p.practice AND
prac.setting = 4

  GROUP BY practice, year_mon ),
```

```
-- select all exem and letro prescribing data, no need to adjust for ADQ
t AS (SELECT practice, bnf_name, items, quantity, month,
        1 AS dosage,
        quantity  AS adq
      FROM ebmdatalab.helen.exemestane_letrozole_all_prescribing ),


-- total exem and letro ADQs and ADQs per 1000 females per practice per month
p AS (SELECT
        CASE WHEN m.code IS NOT NULL THEN 1 ELSE 0 END AS manc_flag,
        q2.practice,
        CAST(q2.year_mon AS DATE) AS month,
        SUM(t.adq) AS adq,
        MAX(q2.female_total) AS female_total,
        MAX(MAX(q2.female_35_44)) OVER (PARTITION BY q2.practice) AS
female_35_44_max,
        1000*IEEE_DIVIDE(sum(t.adq),max(q2.female_total)) AS adq_per_thou
      FROM q2
      LEFT JOIN t ON t.practice = q2.practice AND CAST(t.month AS DATE) =
q2.year_mon
      LEFT JOIN manc m ON  m.code = q2.practice
      GROUP BY  practice, month, manc_flag),


-- Group practices up to show only -/+ Manchester flag
p2 AS (
      SELECT  p.manc_flag,
              p.month,
              SUM(p.adq) AS adq,
              SUM(p.female_total) AS female_total,
              1000*IEEE_DIVIDE(sum(p.adq),sum(p.female_total)) AS adq_perthou
      FROM  p
      WHERE p.month BETWEEN '2010-10-01' AND '2017-10-01'
        AND p.female_35_44_max > 10
      GROUP BY manc_flag, month )

-- results including ratio calculations
SELECT
    p2.manc_flag,
    p2.month,
    p2.adq,
    p2.female_total,
    p2.adq_perthou
FROM p2
ORDER BY month, manc_flag
```

Appendix 3. Stata code used for Interrupted Time Series Analysis

```
cd H:\Stata\
clear
*******************ALL******************
import excel "tamoxifen and raloxifene for ITSA.xls",
sheet("tamoxifen") firstrow clear
drop if month == .
drop if month >= date("2017-01-01","YMD")
gen month2=month(month)
gen tm = mofd(month)
format tm %tmMonCCYY
tsset tm
local date = ym(2013, 06)
xi: itsa adq_perthou i.month2, ///
trperiod(`date') figure(subtitle("") note("") msymbol(X i) ///
xtitle(Date) ytitle(ADQ per thousand) ylabel(,format(%14.0fc))
xlabel(#8,format(%tmMonCCYY)) ///
graphregion(c(white)))
graph export "ITSA-tamoxifen_all_01-2018.png", as(png) replace
*************Greater Manchester**********
import excel "tamoxifen and raloxifene for ITSA.xls", sheet("tamox
2-sample") firstrow clear
drop if month == .
drop if month >= date("2017-01-01","YMD")
drop if month < mdy(04, 30, 2011)
gen month2=month(month)
gen tm = mofd(month)
format tm %tmMonCCYY
local date = ym(2013, 06)
tsset manc_flag tm
xi: itsa adq_perthou i.month2, ///
trperiod(`date') treatid(1) figure(title("") subtitle("") note("")
///
xtitle(Date) ytitle(ADQ per thousand) ylabel(,format(%14.0fc))
xlabel(#8,format(%tmMonCCYY)) ///
graphregion(c(white)))
graph export "ITSA-tamoxifen_GM_01-2018.png", as(png) replace
*******************Raloxifene*****************
import excel "tamoxifen and raloxifene for ITSA.xls", sheet("ralox
2-sample") firstrow clear
```

```
drop if month == .
drop if month >= date("2017-01-01","YMD")
drop if month < mdy(04, 30, 2011)
gen month2=month(month)
gen tm = mofd(month)
format tm %tmMonCCYY
local date = ym(2013, 06)
tsset manc_flag tm
xi: itsa adq_perthou i.month2, ///
trperiod(`date') treatid(1) figure(title("") subtitle("") note("")
///
xtitle(Date) ytitle(ADQ per thousand) ylabel(,format(%14.0fc))
xlabel(#8,format(%tmMonCCYY)) ///
graphregion(c(white)))
graph export "ITSA-raloxifene_01-2018.png", as(png) replace
```
