# Supplementary material for: Impact of NICE guidance on tamoxifen prescribing in England 2011–2017: an interrupted time series analysis
Source: Br J Cancer. 2018 Apr 23;118(9):1268–75. doi: 10.1038/s41416-018-0065-2 (PMC5943266; doi:10.1038/s41416-018-0065-2)
Supplement: Supplementary file 5 — Supplementary materials [file 41416_2018_65_MOESM5_ESM.docx]

**Supplementary materials:**

Appendix 1. Additional Tables and Figures.

Appendix 2. SQL Codes for data extraction.

Appendix 3. Stata code for Interrupted Time Series Analysis.

Appendix 4. Full ITSA input datasets [provided as additional Excel file].

Appendix 5. Full ITSA results datasets [provided as additional Excel file].

Appendix 6. Percentile results datasets [provided as additional Excel file].
